# Long‐term nitrogen input reduces soil bacterial network complexity by shifts in life history strategy in temperate grassland

**DOI:** 10.1002/imt2.194

**Published:** 2024-04-15

**Authors:** Chao Wang, Ziyue Shi, Aogui Li, Tianyi Geng, Lingli Liu, Weixing Liu

**Affiliations:** ^1^ State Key Laboratory of Efficient Utilization of Arid and Semi‐Arid Arable Land in Northern China, Hulunber Grassland Ecosystem National Observation and Research Station, Institute of Agricultural Resources and Regional Planning Chinese Academy of Agricultural Sciences Beijing China; ^2^ State Key Laboratory of Vegetation and Environmental Change, Institute of Botany Chinese Academy of Sciences Beijing China

## Abstract

We investigated soil bacterial and fungal communities, constructed co‐occurrence networks, and estimated bacterial traits along a gradient of nitrogen (N) input. The results showed that soil bacterial co‐occurrence networks complexity decreased with increasing N input. The ratio of negative to positive cohesion decreased with increasing N input, suggesting the declined competitive but strengthened cooperative interactions. However, soil fungal network complexity did not change under N enrichment. In addition, N input stimulated the copiotroph/oligotroph ratio, ribosomal RNA operon (*rrn*) copy number, and guanine‐cytosine (GC) content of soil bacteria, shifting bacterial life history strategy toward copiotroph with increased *r*‐/*K*‐strategy ratio. Piecewise structural equation modeling results further revealed that the reduction in bacterial co‐occurrence network complexity was directly regulated by the increased bacterial *r*‐/*K*‐strategy ratio, rather than reduced bacterial richness. Our study reveals the mechanisms through which microbial traits regulate interactions and shape co‐occurrence networks under global changes.

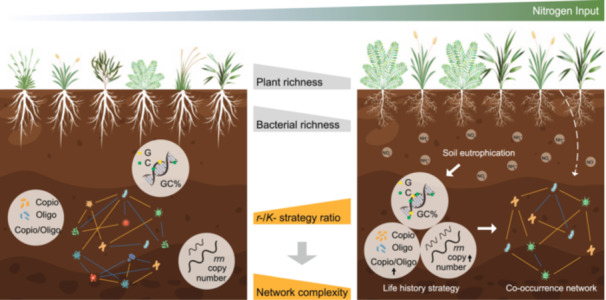

Nitrogen (N) deposition induced by the emission of nitrogenous compounds from the combustion of fossil fuels and the application of N fertilizers has risen by 200% and is projected to double by 2050 in various regions worldwide [1]. The subsequent N enrichment poses serious threats to plant diversity, community, and the functioning of terrestrial ecosystems, particularly grasslands [2]. Ecosystem function is associated with not only changes in plants but also the dynamics of soil microbes in response to N input [3]. Alterations in microbial populations can trigger changes in interactions among microorganisms, which play an important role in maintaining microbial network complexity, stability, and function [4]. Thus, understanding microbial co‐occurrence and interaction is essential in the prediction of ecosystem processes and function in response to N enrichment in grassland ecosystems.

Microbial interaction comprises cooperative and competitive components and generally links to richness. However, a few recent studies of N enrichment on microbial interactions have produced mixed results, irrespective of changes in microbial richness [5, 6, 7]. Specifically, some studies reported an increase in soil bacterial interactions and network complexity following N application, attributing to an increased number of keystone taxa [6]. Conversely, an investigation of a 150‐year manipulative N application showed a significant reduction in the complexity of soil bacterial networks, coupling with a decline in cooperative relationships within communities [5]. Furthermore, one multilevel N application study indicated higher bacterial network complexity under lower N input but diminished complexity under higher N input with the same trend of cooperative relationships [7]. Across those studies, the responses of cooperative relationships to N input were found to align with alterations in bacterial network complexity.

Different from the regulatory effect of cooperation on microbial network complexity, some community ecologists have proposed that the existence of competitive interaction facilitates high‐order interaction and consequently enhances the complexity and stability of co‐occurrence networks [4]. That said, the enhancement of competitive interactions contributes to microbial network complexity. Accordingly, the above observations of declined cooperation with simplified network complexity seem contradictory to this theoretical coexistence conceptual statement between positive (negative) interactions and complexity. Therefore, despite extensive investigations into microbial diversity and communities in grassland ecosystems globally, there is still a severe lack of information regarding how microbial interactions and network complexity respond to N enrichment and their underlying mechanisms.

Here, we investigate microbial co‐occurrence networks along a gradient of manipulative N input in a temperate grassland in China. This study aims to explore how soil microbial interactions respond to N input and elucidate the potential driving factors and mechanisms. We will address the following questions: (1) Whether the reduced microbial richness will lead to the diminution in microbial network complexity under N enrichment? (2) How do cooperative (competitive) interactions change in microbial network complexity in response to N enrichment? (3) Given that soil fungi are more closely connected to plants, will N input exert a more pronounced impact on fungal network complexity?

## RESULTS AND DISCUSSION

**Figure 1:**
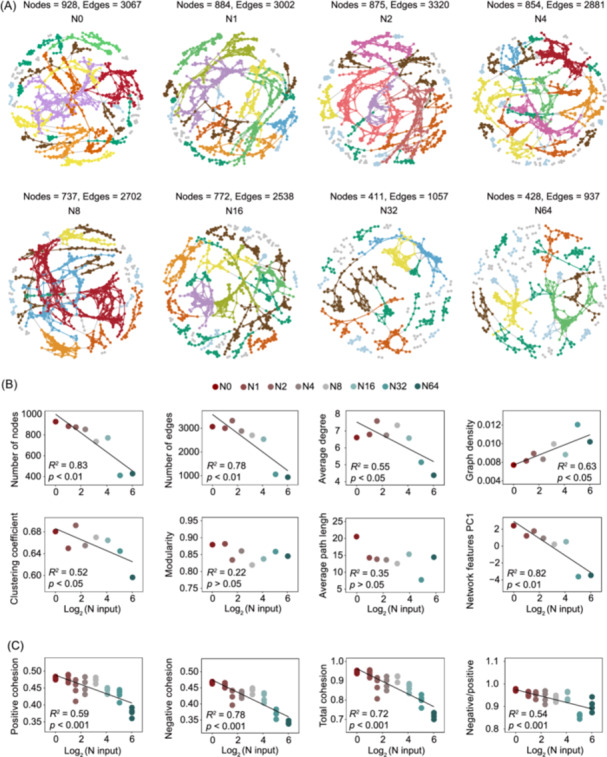
Bacterial co‐occurrence networks, network topology parameters, and cohesion under different nitrogen (N) input treatments. (A) Bacterial co‐occurrence network under different N input treatments. (B) The topological parameters include number of nodes, number of edges, average degree, graph density, clustering coefficient, modularity, average path length, and the first axis of principal component analysis (PC1) value of those network topological parameters. (C) The positive cohesion, absolute value of negative cohesion, total cohesion, and absolute value of negative/positive cohesion of soil bacterial co‐occurrence networks under different N inputs. Nodes indicate individual operational taxonomic units (OTUs), while edges represent significant correlations between OTUs. The colors of nodes are classified according to the number of nodes in the modules. The network features PC1 for principal component analysis of overall topological parameters. The topological parameters include the number of nodes, the number of edges, average degree, graph density, clustering coefficient, modularity, and average path length. Only prominent regression lines are displayed.

**Figure 2:**
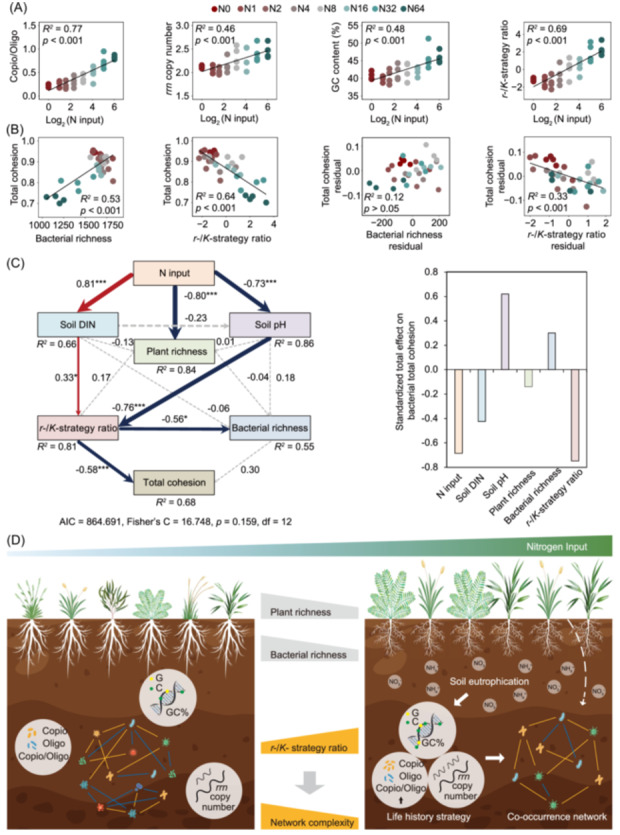
Bacterial life history traits under different nitrogen (N) input treatments and the influencing factors of bacterial network complexity. (A) Soil bacterial copiotroph/oligotroph ratio, ribosomal RNA operon (*rrn*) copy number, guanine‐cytosine (GC) content, and bacterial *r*‐/*K*‐strategy ratio with increasing N input. (B) The relationships between total cohesion and richness and *r*‐/*K*‐strategy ratio, partial regression of total cohesion and bacterial richness or bacterial traits when controlling for bacterial traits or bacterial richness, respectively. (C) The piecewise structural equation modeling (SEM) employed to examine the predictive factors and pathways by which bacterial total cohesion responds to N input. The bacterial *r*‐/*K*‐strategy ratio was created by the first axis of principal component analysis (PC1) value based on the copiotroph/oligotroph ratio, *rrn* copy number, and GC content. The variables of the SEM include soil dissolved inorganic N (DIN), soil pH, plant richness, soil bacterial *r*‐/*K*‐strategy ratio, and bacterial richness. Red and blue solid arrows indicate statistically significant (*p* < 0.05) positive and negative correlations. The width of the red and blue arrows is proportional to the strength of the standardized path coefficient. * and *** indicated *p* < 0.05 and *p* < 0.001, respectively. *R* ^2^ represents the fraction of variance explained. The evaluation parameter of the modeling is Fisher's C (*p* > 0.05). (D) Conceptual illustration depicting how N input influences the complexity of bacterial co‐occurrence networks by mediating bacterial life history strategy, rather than through a reduction in plant or bacterial richness. Soil eutrophication leads to increases in bacterial copiotroph/oligotroph ratio, *rrn* copy number, and GC content, which shifts bacterial community toward a copiotrophic strategy. This shift in bacterial life history strategy contributes to a reduced complexity of bacterial co‐occurrence network. The dashed line means insignificant effects of plant richness on bacterial network. Here, “Copio/Oligo” refers to the copiotroph/oligotroph ratio, and “GC%” refers to the GC content.

The experimental site is located in the semiarid grassland of Duolun County, Inner Mongolia, China. A Latin square design was utilized since 2003, comprising a total 8 N application levels (0, 1, 2, 4, 8, 16, 32, and 64 g N m^−2^ y^−1^). Soil samples were collected in August 2016 and used for sequencing and chemical analyses (see Supporting Information: Methods). With increasing N input, there was a pronounced diminishing trend in the number of nodes and edges, average degree, clustering coefficient, and the first axis of principal component analysis (PC1) of bacterial network characteristics (Figure 1A,B and S1). Similar to topological parameters, the total, positive, negative cohesion, and negative/positive value of bacterial communities decreased with increasing N input (Figure 1C). However, N input did not alter soil fungal network complexity (Figure S2). The copiotroph/oligotroph ratio, ribosomal RNA operon (*rrn*) copy number, guanine‐cytosine (GC) content, and the PC1 value representing the overall bacterial *r*‐/*K*‐strategy ratio gradually increased with the elevated N input (Figure 2A). Total cohesion exhibited a positive correlation with bacterial richness but a negative correlation with the *r*‐/*K*‐strategy ratio (Figure 2B). Piecewise structural equation modeling (SEM) results showed that N input, by increasing soil dissolved inorganic N content (DIN) and decreasing soil pH, altered the bacterial *r*‐/*K*‐strategy ratio. The bacterial *r*‐/*K*‐strategy ratio negatively affected bacterial richness. Total bacterial cohesion had a direct negative association with the *r*‐/*K*‐strategy ratio but had no association with bacterial richness (Figure 2C).

In line with greater *rrn* copy number under high N conditions [8], we found that N input stimulated soil DIN and increased bacterial *rrn* copy number toward the copiotrophic community (Figure S3). We further found that GC content increased by N application. According to the rule of molecular biosynthesis, each GC at base pairs requires 8 N atoms while adenine‐thymine (AT) at base pairs requires only 7 N atoms [9]. Therefore, the “resource‐driven selection” theory proposes that N limitation is a strong selective force that causes the relatively low GC content [10, 11]. Consequently, the greater soil N would result in nucleotide sequences with G+C bias by alleviating N limitation. Different from the previous reports that GC content positively correlated with soil pH at a regional scale [11], GC content negatively related to soil pH in our study (Figure S4). This may be due to a decrease in soil pH caused by extra N application. Overall, a higher bacterial *r*‐/*K*‐strategy ratio indicates that bacterial life history strategy tends toward *r*‐selection strategy. The stimulated *r*‐/*K*‐strategy ratio supports “resource‐driven selection” in bacterial communities in response to N enrichment in the temperate grassland.

The reduced bacterial network complexity under high N input was accompanied by a reduction in richness which was found in our previous work [12] and exhibited a positive correlation (Figure 2B). Note that we found that reduced richness was ascribed to the loss of oligotrophic taxa (Figure S5), which resulted in a shift in life history strategy. When controlling for the *r*‐/*K*‐strategy ratio, the partial correlation of total cohesion with richness is no longer significant (Figure 2B). Furthermore, the SEM results also revealed that bacterial community complexity was negatively associated with the *r*‐/*K*‐strategy ratio, implying that the tightness of bacterial connections was driven by life history strategies rather than bacterial richness (Figure 2B,C and S6). Oligotrophic taxa generally acquire nutrients or energy from various complex recalcitrant matter [13]. This acquisition process consists of multiple metabolic pathways involving various microorganisms, establishing more effective and stronger complex connections among microbial taxa [8]. However, N input stimulates plant growth and consequent fresh carbon inputs (Figure S7), producing sufficient energy supply and labile substrates [14]. The excessive N as well as substrate application facilitates copiotroph and promotes bacterial independence, weakening the complex connections and leading to less connectivity [5], thus simplifying co‐occurrence networks. This finding that the *r*‐/*K*‐strategy ratio mediates bacterial co‐occurrence network complexity points out the regulatory role of bacterial genetic and coding traits in taxa co‐occurrence.

We speculated that the reduced cohesion would be linked to the deconstruction of high‐order interaction in a community. Interaction chains refer to the indirect impacts of one species on another through changes in the abundance of a third species beyond the pairwise interactions [15]. These emerged interaction chains can produce high‐order interactions obtaining more diverse pairwise relationships and maintaining complex and robust co‐occurrence networks [4]. The reduced cohesion and simplified bacterial co‐occurrence network, in turn, to some degree, suggest the deconstruction of high‐order interactions as well as a shift toward low‐order interactions under N enrichment.

The ratio between negative and positive cohesion represents the changes in competitive or cooperative strength. A higher ratio indicates greater competition among taxa within the community. Opposite to the increased negative/positive ratio in typical grassland [7], our results showed a decreased negative/positive cohesion ratio with increasing N input. This indicates a diminishment of competition and reinforcement of cooperation within the community. Specifically, extra N input directly alleviates the competition for nutrients and could weaken the competitive interactions [6]. In addition, the fast growth and metabolism within diverse taxa will produce metabolites to facilitate the exchange of energy and materials among taxa called cross‐feeding to enhance cooperation [16]. Correspondingly, we found enhanced cooperation with an increased bacterial *r*‐/*K*‐strategy ratio (Figure S8). Moreover, certain competition can promote coexistence by equalizing the fitness of the other taxa within the community [17]. The loss of such competitors may lead to the subsequent loss of others and a simplified co‐occurrence network [15]. The concurrent decreasing competitive interaction and simplified network emphasize the role of competition in maintaining species coexistence.

The finding of unchanged soil fungal co‐occurrence network complexity did not support our third hypothesis that soil fungi would be more responsive to N input. The unchanged co‐occurrence network could be attributed to the physiological and morphological properties of soil fungi. Soil fungi generally have a great capacity to mineralize complex organic compounds for N [18]. Moreover, they also have high N use efficiency in biomass accumulation and a high C/N ratio [19]. Those two characteristics facilitate soil fungal growth under low N conditions with less dependence on extra N input, manifesting insensitive to N input. Furthermore, the thick cell walls and intrinsic spores exhibit high tolerance to soil acidification and metal toxicity [18], remaining stable under N enrichment. The feature of fungal mycelium enables it to penetrate and spread underground to establish a hyphal network [20]. Consequently, soil fungal hyphal network can extend over a wider spatial distribution and is therefore resistant to environmental change.

## CONCLUSION

In conclusion, our study demonstrates distinct patterns of changes in bacterial and fungal networks and interactions along the gradient of increasing N input in a temperate steppe. As N input increased, bacterial network complexity exhibited a downward trend, accompanied by a decline in the negative/positive cohesion ratio, while the network complexity of fungal communities remained unaltered. We further found that the decline in bacterial network complexity was driven by the increase in bacterial *r*‐/*K*‐strategy ratio under N enrichment. It is worth noting that this result is derived from a study over a decade of N input. Given the cumulative effect of N application over time, the temporal dynamics of soil bacterial network complexity in response to N input should be considered when extrapolating. Nevertheless, to the best of our knowledge, this study is the first empirical report that the reduced bacterial complexity is predominantly ascribed to the changes in bacterial life history strategy traits under N enrichment in grassland ecosystems. This finding provides insights into a more comprehensive understanding of the underlying mechanisms by which microbial traits modulate co‐occurrence networks and interactions in response to environmental changes.

## AUTHOR CONTRIBUTIONS

Weixing Liu conceived the idea and supervised the study. Chao Wang and Weixing Liu analyzed the data. Chao Wang and Weixing Liu wrote the manuscript with input from all authors. Ziyue Shi, Aogui Li, Tianyi Geng, and Lingli Liu made contributions to the revision of the manuscript. All authors have read the final manuscript and approved it for publication.

## CONFLICT OF INTEREST STATEMENT

The authors declare no conflict of interest.

## ETHICS STATEMENT

No animals or humans were involved in this study.

## Supporting information


**Figure S1**: The edges in bacterial networks under different nitrogen (N) input treatments.
**Figure S2**: Fungal co‐occurrence networks and network topology parameters under different nitrogen (N) input treatments.
**Figure S3**: Effects of soil dissolved inorganic nitrogen (DIN) on bacterial life history traits.
**Figure S4**: Effects of soil pH on bacterial life history traits.
**Figure S5**: The bacterial operational taxonomic unit (OTU) richness of different trophic types with increasing nitrogen (N) input.
**Figure S6**: Effects of bacterial life history traits on total cohesion in bacterial co‐occurrence networks.
**Figure S7**: Aboveground biomass with increasing nitrogen (N) input.
**Figure S8**: Effects of bacterial life history traits on negative/positive cohesion in bacterial co‐occurrence networks.

## Data Availability

(The 16S and ITS sequences were submitted to the NCBI under accession numbers PRJNA573484 http://www.ncbi.nlm.nih.gov/search/all/?term=PRJNA573484) and PRJNA573488 (http://www.ncbi.nlm.nih.gov/search/all/?term=PRJNA573488). The data, scripts, and Table S used are saved in GitHub (http://github.com/ChaoWang-01/code-for-imeta). Supplementary materials (methods, figures, scripts, graphical abstract, slides, videos, Chinese translated version, and update materials) can be found in the online DOI or iMeta Science http://www.imeta.science/.
