# Supplementary material for: Long‐term nitrogen input reduces soil bacterial network complexity by shifts in life history strategy in temperate grassland
Source: Imeta. 2024 Apr 15;3(3):e194. doi: 10.1002/imt2.194 (PMC11183191; doi:10.1002/imt2.194)
Supplement: Supplementary file 1 — Figure S1: The edges in bacterial networks under different nitrogen (N) input treatments. Figure S2: Fungal co‐occurrence networks and network topology parameters under different nitrogen (N) input treatments. Figure S3: Effects of soil dissolved inorganic nitrogen (DIN) on bacterial life history traits. Figure S4: Effects of soil pH on bacterial life history traits. Figure S5: The bacterial operational taxonomic unit (OTU) richness of different trophic types with increasing nitrogen (N) input. Figure S6: Effects of bacterial life history traits on total cohesion in bacterial co‐occurrence networks. Figure S7: Aboveground biomass with increasing nitrogen (N) input. Figure S8: Effects of bacterial life history traits on negative/positive cohesion in bacterial co‐occurrence networks. [file IMT2-3-e194-s001.docx]

**Supporting information to:**

**Long-term nitrogen input reduces soil bacterial network complexity by shifts in life-history strategy in temperate grassland**

**Running Title:** Soil bacterial traits mediate network complexity under nitrogen enrichment

Chao Wang^1^, Ziyue Shi^1^, Aogui Li^1^, Tianyi Geng^1^, Lingli Liu^2^, Weixing Liu^1^*

^1^State Key Laboratory of Efficient Utilization of Arid and Semi-arid Arable Land in Northern China, National Hulunber Grassland Ecosystem Observation and Research Station, Institute of Agricultural Resources and Regional Planning, Chinese Academy of Agricultural Sciences, Beijing 100081, China

^2^State Key Laboratory of Vegetation and Environmental Change, Institute of Botany, Chinese Academy of Sciences, Beijing 100093, China

*Correspondence: [liuweixing@caas.cn](mailto:liuweixing@caas.cn) (Weixing Liu)

**METHODS**

**Experimental design**

The experimental site is located in the semi-arid grassland of Duolun County, Inner Mongolia, China (42.01′ N, 116.16′ E, 1324 m altitude). The area is affected by monsoons, with cold and dry winters, and warm and humid summers. The annual average temperature is 2.1 ℃, and the monthly average temperature is -17.5 ℃ (January) to 18.9 ℃ (July). The annual average precipitation is 382.3 mm, about 50% in July and August. According to the classification of the Food and Agriculture Organization of the United Nations, the soil type is Haplic Calcisols, containing 69.21 ± 0.06% sand, 15.60 ± 0.02% silt, and 15.19 ± 0.02% clay. The soil organic carbon and total nitrogen (N) concentrations are 16.94 ± 2.34 g kg^−1^ and 1.65 ± 0.27 g kg^−1^, respectively, the soil pH is 6.84 ± 0.02, and the environmental N deposition was approximately 1.6 g N m^−2^ [1]. The plant community is dominated by perennial herbs and grasses, including *Stipa krylovii* Roshev*.*, *Agropyron cristatum* L., *Artemisia frigida* Willd and *Cleistogenes squarrosa* Trin..

N fertilization experiment has been carried out since 2003. A Latin square design was utilized, comprising 64 experimental plots with a size of 10 × 15 m. These plots were arranged in 8 rows and 8 columns, with a 5 m buffer zone between adjacent plots. Each row with 8 plots was randomly assigned to one of 8 N application levels (0, 1, 2, 4, 8, 16, 32, and 64 g N m^−2^ y^−1^). Annually, N is applied in the form of urea in early July. Since 2005, four rows (one row in every two rows) were mowed on the ground in late August, and all hay in the experiment plots were removed. In this study, samples were collected exclusively from unmown plots.

**Measurement of soil chemical properties and plants**

Soil samples were collected on August 15, 2016. A soil auger was used to randomly collect 6 drilled soil cores (15 cm deep, 5 cm diameter) in each plot and mix them into one sample. After sieving roots and stones through a 2 mm mesh, soil samples were stored in ice boxes and transferred to the laboratory immediately. The soil samples were divided into two subsamples, one subsample was stored in a 4 °C refrigerator for soil physicochemical analysis, and the other was stored in a -80 °C refrigerator for DNA extraction. Total soil N was determined using elemental analysis (Elementar Analysensysteme GmbH). Soil pH was measured using a glass electrode on a soil-to-water ratio suspension of 1:2.5. Soil dissolved inorganic N (DIN) was extracted using 2 M KCl solution, and NH_4_^+^ and NO_3_^−^ were measured using a flow injection analyzer.

The point-intercept method was used to investigate the plant communities at the peak biomass within the 1 × 1 m permanent quadrat in each plot. Plant species richness was estimated by placing a 1 × 1 m frame and 100 evenly spaced 10 × 10 cm grids on permanent quadrats. Then plant species richness for each experimental plot was recorded as the number of plant species present in the quadrat.

**Amplicon sequencing**

Soil DNA was extracted using the PowerSoil DNA isolation kit (MoBio Laboratories), weighing 0.5 g of fresh soil per sample and following the manufacturer's instructions. The bacterial community used primers 338F (5′-ACTCCTACGGGAGGCAGCAG-3′) and 806R (5′-GGACTACHVGGGTWTCTAAT-3′) to amplify the V3-V4 hypervariable region of the 16S rRNA gene [2]. The fungal community amplified the Internal Transcribed Spacer 1 (ITS1) region using ITS1-F (5′-CTTGGTCATTTAGAGGAAGTAA-3′) and ITS2 (5′-TGCGTTTCTTCATCGATGC-3′) [3]. A unique 10 bp barcode for each sample is attached to the 5 'end of the primer to distinguish each sample. The 50 μL PCR reaction system included 5 μL 10× Ex Taq Buffer (Mg^2+^ plus), 4 μL 12.5 mM dNTP Mix, 1.25 U Ex Taq DNA polymerase (TaKaRa), 2 μL template DNA, and 36.75 μL ddH_2_O. The PCR mixture was pre-denatured at 94 °C for 2 min, followed by 30 cycles of amplification (94 °C for 30 s, 57 °C for 30 s, and 72 °C for 30 s), and finally extended at 72 °C for 10 min. PCR was performed three times on each sample, and each batch had a negative control. PCR products were mixed at equimolar concentrations and purified using QIAquick Gel Extraction Kit (Qiagen). Purified PCR products were sequenced on the Illumina MiSeq platform (Illumina).

Raw reads were trimmed and low-quality bases (Phred score < 20) were removed. Sequence splitting was performed using FLASH software (version 1.0.0) based on the unique barcode of each sample [4]. Barcodes and primers were removed from these sequences using MOTHUR software [5]. Then, the sequences that do not meet the criteria are excluded, such as those containing ambiguous bases or sequences with a length of less than 200 bp. The remaining sequences were clustered into operational taxonomic units (OTUs) with 97% similarity using the UPARSE algorithm [6], removing chimeras in the process. The Ribosomal Database Project (RDP) classifier [7] was used to assign 16S rRNA and ITS gene sequences to taxonomic groups based on the SILVA 128 database [8] and the UNITE fungal ITS 7.2 database [9], and the identification threshold was 70%. Simultaneously, unclassified bacterial and fungal OTUs were also removed. And rarefied all samples of bacteria (18,877) and fungi (24,945) to the same depth respectively. All sequences have been deposited in NCBI’s SRA database, and the project accession numbers are PRJNA573484 and PRJNA573488.

**Data analyses**

All statistical analyses were conducted using R 4.3.1, and data visualizations were performed using “ggplot2” package [10]. Co-occurrence networks for bacterial and fungal communities were constructed under each N input treatment respectively. Within the same N input treatment, only OTUs with a relative abundance greater than 0.01% and present in all samples are included in the network [11]. The Pearson coefficient was calculated to determine the correlation between OTUs. These measured correlations were used to represent the edges in the network [12]. To minimize the uncertainty in network construction and comparison, the “RMThreshold” package was used to determine the microbial network construction threshold according to the random matrix theory. A threshold value of 0.75 was identified and the edges with *p* values less than 0.01 were selected [13].

Microbial interactions and complexity are assessed by constructing complex co-occurrence networks containing positive and negative correlations that represent cooperative and competitive behaviors between coexisting taxa [14–16]. The number of nodes in a network represents the number of species that exist in the network. The “igraph” package was performed to visualize the co-occurrence network of bacterial and fungal communities and compute network topological parameters including the number of nodes, edges, average degree, clustering coefficient, and modularity [17]. These parameters were applied to evaluate the complexity of the co-occurrence network [12]. Bivariate linear regression was used to analyze the relationship between topological parameters of bacterial communities as well as fungal communities and N input levels. The amount of N input was log-transformed. Principal component analysis was performed on topological parameters and then the first axis of principal component analysis (PC1) values were used to represent overall bacterial and fungal network properties and complexity. Positive and negative correlation edges, along with the proportion of positive correlation edges to the total number of edges in the co-occurrence network, were extracted and subjected to regression analysis with N input levels.

Cohesion was calculated to indicate the complexity of bacterial and fungal communities based on the previous method [12]. The formula is as follows:

$$cohesion=\sum_{i=1}^{n} {abundance}_{i} \times{connectedness}_{i}$$

where *n* represents the total number of taxa groups in the community. Positive and negative cohesion values were measured for each sample by summing the significant positive and negative correlations between taxa, weighted by their respective abundance [18,19]. The values of negative and positive cohesion range from -1 to 0 and 0 to 1, respectively. Higher absolute total cohesion values indicate more correlations and greater absolute negative cohesion values indicate more stability. Total cohesion was determined by summing the positive and absolute negative values. The ratio between negative and positive cohesion was measured using absolute values to represent the changes in competitive or cooperative strength. Bivariate linear regression was also used to analyze the relationship between cohesion indices and N input levels.

Bacterial traits associated with life history were quantified based on three parameters. Copiotrophic (*r*-strategy) and oligotrophic (*K*-strategy) bacteria were identified according to the existing classification of life history strategy. Specifically, Alpha- and Delta-Proteobacteria, Planctomycetes, and Acidobacteria can mineralize recalcitrant carbon as the growth of caborn resource and energy [20–22], Chloroflexi exhibit a slow growth rate, thriving in soils with poor nutrients [20–23]. All these taxa were classified as oligotrophic bacteria with K-strategy. Conversely, Beta- and Gamma-Proteobacteria, and Bacteroidetes were defined as copiotrophs due to the utilization of labile C substrates [24,25]. In addition, Firmicutes were also regarded as copiotrophs owing to the decomposition of fresh organic matter [26]. The proportion of the abundance of those two strategists (copiotroph/oligotroph) was calculated to represent changes in the bacterial life history strategy.

Bacterial average ribosomal RNA operon (*rrn*) copy number at community level was estimated according to the *rrn*DB database (version 5.8, https://rrndb. umms.med.umich.edu/) [27]. We matched the *rrn* copy number starting from lowest rank (species). If there was no match at the lowest level, the average *rrn* copy number of the higher level was used [28]. The community-level *rrn* copy number was then calculated by the abundance-weighted mean *rrn* copy number of all OTUs.

Bacterial average GC content at the community level was calculated by annotating 16S rRNA representative sequence to the Genome Taxonomy Database (GTDB) [29]. We downloaded the FASTA file (<https://data.gtdb.ecogenomic.org/releases/latest/genomic_files_reps/bac120_ssu_reps.tar.gz>)of 16S rRNA gene sequences identified within the set of bacterial representative genomes and converted it into a reference database by makeblastdb tool [30]. BLAST (version 2.15.0) was used to align 16S representative sequences with reference databases at ≥ 97% identity. After obtaining the accession ID, GC content was matched to the metadata file downloaded in GTDB (<https://data.gtdb.ecogenomic.org/releases/latest/bac120_metadata.tsv.gz>). The community-level GC content was then calculated by the abundance-weighted mean GC content of all OTUs. Principal component analysis was then performed based on the above three traits and then PC1 values were used to represent the overall bacterial *r*-/*K*-strategy ratio.

Piecewise structural equation modeling (SEM) was applied to explore the direct and indirect pathways that N input regulates bacterial traits and network complexity using “piecewiseSEM” packages [31]. This model proposed the hypothesized causal linkages among soil properties, plant richness, bacterial richness, and traits. The bacterial traits were quantified as *r*-/*K*-strategy ratio based on the copiotroph/oligotroph ratio, the *rrn* copy number, and GC content. Fisher’s C statistic was used to evaluate the goodness of fit of SEM. Non-important paths were successively removed from the initial SEM, and the final structure was selected based on the lowest Akaike Information Criterion (AIC).

**REFERENCES**

1. Liu, Weixing, Chunlian Qiao, Sen Yang, Wenming Bai, Lingli Liu. 2018. “Microbial carbon use efficiency and priming effect regulate soil carbon storage under nitrogen deposition by slowing soil organic matter decomposition.” *Geoderma* 332: 37-44. <https://doi.org/10.1016/j.geoderma.2018.07.008>

2. Caporaso, J. Gregory, Christian L. Lauber, William A. Walters, Donna Berg-Lyons, James Huntley, Noah Fierer, Sarah M. Owens, et al. 2012. “Ultra-high-throughput microbial community analysis on the Illumina HiSeq and MiSeq platforms.” *The ISME Journal* 6: 1621-1624. <https://doi.org/10.1038/ismej.2012.8>

3. McGuire, Krista L., Sara G. Payne, Matthew I. Palmer, Caitlyn M. Gillikin, Dominique Keefe, Su Jin Kim, Seren M. Gedallovich, et al. 2013. “Digging the new york city skyline: soil fungal communities in green roofs and city parks.” *PLoS One* 8: e58020. <https://doi.org/10.1371/journal.pone.0058020>

4. Magoč, Tanja, Steven L. Salzberg. 2011. “FLASH: fast length adjustment of short reads to improve genome assemblies.” *Bioinformatics* 27: 2957-2963. <https://doi.org/10.1093/bioinformatics/btr507>

5. Schloss Patrick, D., L. Westcott Sarah, Thomas Ryabin, R. Hall Justine, Martin Hartmann, B. Hollister Emily, A. Lesniewski Ryan, et al. 2009. “Introducing mothur: open-source, platform-independent, community-supported software for describing and comparing microbial communities.” *Applied and Environmental Microbiology* 75: 7537-7541. <https://doi.org/10.1128/AEM.01541-09>

6. Edgar, R. C. 2013. “UPARSE: highly accurate OTU sequences from microbial amplicon reads.” *Nature Methods* 10: 996-998. <https://doi.org/10.1038/nmeth.2604>

7. Wang, Qiong, M. Garrity George, M. Tiedje James, R. Cole James. 2007. “Naive bayesian classifier for rapid assignment of rRNA sequences into the new bacterial taxonomy.” *Applied and Environmental Microbiology* 73: 5261-5267. <https://doi.org/10.1128/AEM.00062-07>

8. Quast, Christian, Elmar Pruesse, Pelin Yilmaz, Jan Gerken, Timmy Schweer, Pablo Yarza, Jörg Peplies, Frank Oliver Glöckner. 2013. “The SILVA ribosomal RNA gene database project: improved data processing and web-based tools.” *Nucleic Acids Research* 41: D590-D596. <https://doi.org/10.1093/nar/gks1219>

9. Nilsson, Rolf Henrik, Karl-Henrik Larsson, Andy F S Taylor, Johan Bengtsson-Palme, Thomas S. Jeppesen, Dmitry Schigel, Peter Kennedy, et al. 2019. “The UNITE database for molecular identification of fungi: handling dark taxa and parallel taxonomic classifications.” *Nucleic Acids Research* 47: D259-D264. <https://doi.org/10.1093/nar/gky1022>

10. Wickham, Hadley 2016. ggplot2: Elegant graphics for data analysis. <https://doi.org/10.1007/978-3-319-24277-4>

11. Ma, Bin, Haizhen Wang, Melissa Dsouza, Jun Lou, Yan He, Zhongmin Dai, Philip C. Brookes, Jianming Xu, Jack A. Gilbert. 2016. “Geographic patterns of co-occurrence network topological features for soil microbiota at continental scale in eastern China.” *The ISME Journal* 10: 1891-1901. <https://doi.org/10.1038/ismej.2015.261>

12. Yuan, Mengting Maggie, Xue Guo, Linwei Wu, Ya Zhang, Naijia Xiao, Daliang Ning, Zhou Shi, et al. 2021. “Climate warming enhances microbial network complexity and stability.” *Nature Climate Change* 11: 343-348. <https://doi.org/10.1038/s41558-021-00989-9>

13. Deng, Ye, Yi-Huei Jiang, Yunfeng Yang, Zhili He, Feng Luo, Jizhong Zhou. 2012. “Molecular ecological network analyses.” *BMC Bioinformatics* 13: 113. <https://doi.org/10.1186/1471-2105-13-113>

14. Fuhrman, Jed A. 2009. “Microbial community structure and its functional implications.” *Nature* 459: 193-199. <https://doi.org/10.1038/nature08058>

15. Banerjee, Samiran, Florian Walder, Lucie Büchi, Marcel Meyer, Alain Y. Held, Andreas Gattinger, Thomas Keller, Raphael Charles, Marcel G. A. van der Heijden. 2019. “Agricultural intensification reduces microbial network complexity and the abundance of keystone taxa in roots.” *The ISME Journal* 13: 1722-1736. <https://doi.org/10.1038/s41396-019-0383-2>

16. Palmer, Jacob D., Kevin R. Foster. 2022. “Bacterial species rarely work together.” *Science* 376: 581-582. <https://doi.org/10.1126/science.abn5093>

17. Csárdi, Gábor, Nepusz Tamás. 2006. “The igraph software package for complex network research.” *InterJournal, Complex Systems* 1695: 1-9. <http://igraph.sf.net>

18. Herren, Cristina M., Katherine D. McMahon. 2017. “Cohesion: a method for quantifying the connectivity of microbial communities.” *The ISME Journal* 11: 2426-2438. <https://doi.org/10.1038/ismej.2017.91>

19. Hernandez, Damian J., Aaron S. David, Eric S. Menges, Christopher A. Searcy, Michelle E. Afkhami. 2021. “Environmental stress destabilizes microbial networks.” *The ISME Journal* 15: 1722-1734. <https://doi.org/10.1038/s41396-020-00882-x>

20. Barret, Matthieu, John P. Morrissey, Fergal O’Gara. 2011. “Functional genomics analysis of plant growth-promoting rhizobacterial traits involved in rhizosphere competence.” *Biology and Fertility of Soils* 47: 729-743. <https://doi.org/10.1007/s00374-011-0605-x>

21. Ali, Rana Shahbaz, Christian Poll, Ellen Kandeler. 2018. “Dynamics of soil respiration and microbial communities: Interactive controls of temperature and substrate quality.” *Soil Biology and Biochemistry* 127: 60-70. <https://doi.org/10.1016/j.soilbio.2018.09.010>

22. Razanamalala, Kanto, Tantely Razafimbelo, Pierre-Alain Maron, Lionel Ranjard, Nicolas Chemidlin, Mélanie Lelièvre, Samuel Dequiedt, et al. 2018. “Soil microbial diversity drives the priming effect along climate gradients: a case study in Madagascar.” *The ISME Journal* 12: 451-462. <https://doi.org/10.1038/ismej.2017.178>

23. Davis, Kathryn E. R., Parveen Sangwan, Peter H. Janssen. 2011. “Acidobacteria, Rubrobacteridae and Chloroflexi are abundant among very slow-growing and mini-colony-forming soil bacteria.” *Environmental Microbiology* 13: 798-805. <https://doi.org/10.1111/j.1462-2920.2010.02384.x>

24. Fierer, Noah, Mark A. Bradford, Robert B. Jackson. 2007. “Toward an ecological classification of soil bacteria.” *Ecology* 88: 1354-1364. <https://doi.org/10.1890/05-1839>

25. Eilers, Kathryn G., Christian L. Lauber, Rob Knight, Noah Fierer. 2010. “Shifts in bacterial community structure associated with inputs of low molecular weight carbon compounds to soil.” *Soil Biology and Biochemistry* 42: 896-903. <https://doi.org/10.1016/j.soilbio.2010.02.003>

26. Li, Hui, YongYong Zhang, Shan Yang, Zhirui Wang, Xue Feng, Heyong Liu, Yong Jiang. 2019. “Variations in soil bacterial taxonomic profiles and putative functions in response to straw incorporation combined with N fertilization during the maize growing season.” *Agriculture, Ecosystems & Environment* 283: 106578. <https://doi.org/10.1016/j.agee.2019.106578>

27. Stoddard, Steven F., Byron J. Smith, Robert Hein, Benjamin R. K. Roller, Thomas M. Schmidt. 2015. “rrnDB: improved tools for interpreting rRNA gene abundance in bacteria and archaea and a new foundation for future development.” *Nucleic Acids Research* 43: D593-D598. <https://doi.org/10.1093/nar/gku1201>

28. Dai, Tianjiao, Donghui Wen, Colin T. Bates, Linwei Wu, Xue Guo, Suo Liu, Yifan Su, Jiesi Lei, Jizhong Zhou, Yunfeng Yang. 2022. “Nutrient supply controls the linkage between species abundance and ecological interactions in marine bacterial communities.” *Nature Communications* 13: 175. <https://doi.org/10.1038/s41467-021-27857-6>

29. Parks, Donovan H., Maria Chuvochina, Christian Rinke, Aaron J. Mussig, Pierre-Alain Chaumeil, Philip Hugenholtz. 2022. “GTDB: an ongoing census of bacterial and archaeal diversity through a phylogenetically consistent, rank normalized and complete genome-based taxonomy.” *Nucleic Acids Research* 50: D785-D794. <https://doi.org/10.1093/nar/gkab776>

30. Wang, Cong, Qing-Yi Yu, Niu-Niu Ji, Yong Zheng, John W. Taylor, Liang-Dong Guo, Cheng Gao. 2023. “Bacterial genome size and gene functional diversity negatively correlate with taxonomic diversity along a pH gradient.” *Nature Communications* 14: 7437. <https://doi.org/10.1038/s41467-023-43297-w>

31. Lefcheck, Jonathan S. 2016. “piecewiseSEM: Piecewise structural equation modelling in r for ecology, evolution, and systematics.” *Methods in Ecology and Evolution* 7: 573-579. <https://doi.org/10.1111/2041-210X.12512>

**Supplementary figures**

**Figure S1** **The edges in bacterial networks under different nitrogen (N) input treatments.** (A) The positively correlated edges and (B) negatively correlated edges within soil bacterial co-occurrence networks with increasing N input. Only significant regression lines are shown.

**Figure S2 Fungal co-occurrence networks, network topology parameters under different nitrogen (N) input treatments.** (A) Fungal co-occurrence network under different N input treatments; (B) the topological parameters include number of nodes, number of edges, average degree, graph density, clustering coefficient, modularity, average path length and the first axis of principal component analysis (PC1) value of those network topological parameters. Nodes indicate individual operational taxonomic units (OTUs), while edges represent significant correlations between OTUs. The colors of nodes are classified according to the number of nodes in the modules. The network features PC1 for principal component analysis of overall topological parameters. The topological parameters include the number of nodes, the number of edges, average degree, graph density, clustering coefficient, modularity, and average path length. Only prominent regression lines are displayed.

**Figure S3 Effects of soil dissolved inorganic nitrogen (DIN) on bacterial life history traits.** (A) Linear regressions between DIN with the copiotroph/oligotrophy ratio, (B) ribosomal RNA operon (*rrn*) copy number, (C) guanine-cytosine (GC) content, and (D) the first axis of principal component analysis (PC1) value of those three parameters, respectively.

**Figure S4 Effects of soil pH on bacterial life history traits.** (A) Linear regressions between pH with the copiotroph/oligotrophy ratio, (B) ribosomal RNA operon (*rrn*) copy number, (C) guanine-cytosine (GC) content, and (D) the first axis of principal component analysis (PC1) value of those three parameters, respectively.

**Figure S5 The bacteria operational taxonomic unit (OTU) richness of different nutrient types with increasing nitrogen (N) input.** (A) The copiotrophic and (B) oligotrophic bacterial OTU richness with increasing nitrogen input.

**Figure S6 Effects of bacterial life history traits on total cohesion in bacterial co-occurrence networks.** (A) Linear regressions between bacterial total cohesion with the copiotroph/oligotrophy ratio, (B) ribosomal RNA operon (*rrn*) copy number, (C) guanine-cytosine (GC) content, and (D) the first axis of principal component analysis (PC1) value (*r*-/*K*-strategy ratio) of those three parameters, respectively.

**Figure S7 Aboveground biomass with increasing N input.**

**Figure S8 Effects of bacterial life history traits on negative/positive cohesion in bacterial co-occurrence networks.** (A) Linear regressions between the absolute value of negative/positive cohesion in bacterial co-occurrence networks and the abundance of copiotroph/oligotroph ratio, (B) ribosomal RNA operon (*rrn*) copy number, (C) guanine-cytosine (GC) content, and (D) the first axis of principal component analysis (PC1) value (*r*-/*K*-strategy ratio) of those three parameters, respectively.
